# CD20 + T lymphocytes in isolated Hashimoto’s thyroiditis and type 3 autoimmune polyendocrine syndrome: a pilot study

**DOI:** 10.1007/s40618-024-02370-x

**Published:** 2024-04-20

**Authors:** Ilaria Stramazzo, Giorgio Mangino, Silvia Capriello, Giovanna Romeo, Silvia Martina Ferrari, Poupak Fallahi, Maria Flavia Bagaglini, Marco Centanni, Camilla Virili

**Affiliations:** 1 Department of Medico-Surgical Sciences and Biotechnologies, Sapienza University of Rome, Latina, Italy; 2 Department of Clinical and Experimental Medicine, University of Pisa, Pisa, Italy; 3 Department of Translational Research and New Technologies in Medicine and Surgery, University of Pisa, Pisa, Italy; 4 Department of Experimental Medicine, Sapienza University of Rome, Rome, Italy

**Keywords:** CD20^+^ T lymphocytes, Hypothyroidism, Hashimoto’s thyroiditis, Poly-autoimmunity

## Abstract

**Background:**

CD20^+^ T cells represent up to 5% of circulating T lymphocytes. These cells have been shown to produce higher levels of IL-17A and IFN-γ than those of CD20^−^ T lymphocytes. Some reports described the role of CD20^+^ T cells in autoimmune disorders such as multiple sclerosis and rheumatoid arthritis possibly due to their ability to produce these inflammatory cytokines. This study is aimed at describing the behavior of CD20^+^ T lymphocytes in the most frequent autoimmune disorder, i.e., Hashimoto’s thyroiditis (HT), presenting isolated or associated to further autoaggressive disorders in a frame of poly-autoimmunity.

**Methods:**

The study group encompasses 65 HT patients: 23 presenting in isolated form (IT) and 42 with an associated non-endocrine autoimmune disorder [16 with chronic atrophic gastritis (CAG), 15 with nonsegmental vitiligo (VIT), and 11 with celiac disease (CD)]. Twenty healthy donors act as control group (HD). Chronic use of interfering drugs, severe or chronic disorders, and pregnancy and lactation were used as exclusion criteria. Whole blood samples (100 µl) were stained with fluorescent-labeled antibodies (anti-CD45, anti-CD3, anti-CD19, anti-CD16, anti-CD56, anti-CD4, anti-CD8, anti-CD20). Red blood cells were then lysed by adding 1 ml of hypotonic buffer, and samples were acquired on a Flow Cytometer.

**Results:**

CD3^+^CD8^+^CD20^+^ T lymphocytes’ percentages, were significantly higher in the whole group of autoimmune patients compared to healthy donors (*p* = 0.0145). Dividing HT patients based on the type of presentation of autoimmune thyroiditis, CAG group showed the highest percentage of these cells as compared to HD and CD (*p* = 0.0058). IT patients showed higher percentages of CD3^+^ CD8^+^CD20^+^ cells than those of HD patients although not reaching statistical significance. However, dividing IT group based on thyroid function, hypothyroid patients showed higher CD8^+^CD20^+^ cell percentages than those of HD and euthyroid patients (*p* = 0.0111). Moreover, in IT patients, these cells were negatively correlated with FT4 levels (*p* = 0.0171; *r* = −0.4921).

**Conclusions:**

These preliminary findings indicate that CD8^+^CD20^+^ T cells are activated in patients with autoimmune thyroiditis and may behave differently according to the presence of poly-autoimmunity and hypothyroidism.

## Introduction

The non-glycosylated protein CD20 is an immune phenotypic marker associated with B lymphocyte population.^1^ However, it has been described a subset of T lymphocytes expressing this surface antigen, as well.^2^ Indeed, up to 5% of circulating T lymphocytes are CD20 positive.^3^ Neither the origin nor the role of these cells has been fully elucidated so far. Compared to classical T lymphocytes, CD20^+^ is more expressed on CD8 and CD45RO rather than on CD4 T lymphocytes.^4^ Furthermore, CD20^+^ T cells produce higher amounts of proinflammatory cytokines (IL17, TNFα, IFNγ) than canonical T lymphocytes. These cells therefore exhibit phenotypic features of effector and memory cells.^3–4^ The role of CD20^+^ T cells in autoimmune, infectious, and neoplastic diseases has been investigated.^5^ In many autoimmune disorders (rheumatoid arthritis, multiple sclerosis, Sjogren’s syndrome, and psoriasis), these cells have shown quantitative alterations such as an increased frequency and qualitative alterations such as a constitutive cytokine production.^6–9^ Moreover, some studies have been focused on the behavior of these cells in patients treated with anti-CD20 monoclonal antibodies (mAbs).^9–12^ Indeed, these mAbs have been proven effective in reducing these cells in patients with Sjogren’s syndrome ^9^ or with rheumatoid arthritis (RA) (rituximab) ^10–11^ and with multiple sclerosis (ocrelizumab) ^12^ as well. The concomitant action of these drugs on CD20^+^ B cells, however, prevented the authors from correlating the depletion of CD20^+^ T cells with clinical response. The role of CD20^+^ T lymphocytes has been scarcely investigated in thyroid autoimmunity, but in a study carried out some 10 years ago in patients with Graves’s orbitopathy and treated with Rituximab, the authors correlated the reduction of IGF1R^+^ T cells to clinical improvement.^13^ However, whether these cells were also CD20 positive was unknown. Since then, several authors reported the effect of rituximab in patients with Graves’s orbitopathy, which was also quoted in the recent ATA/ETA guideline on thyroid eye disease.^14–17^ Noticeably, a recent study in a murine model of multiple sclerosis showed that the exclusive depletion of CD20^+^ T cells effectively improved the inflammatory process, independently from the B cell involvement.^18^ Based on these recent data and the putative role of CD20^+^ lymphocytes in various autoimmune disorders ^19–20^, the aim of this study was to analyze whether the subset of CD20^+^ T cells is also modulated in the most frequent autoimmune disease, Hashimoto’s thyroiditis, occurring either isolated or associated with non-segmental vitiligo, atrophic gastritis, and celiac disease.

## Materials and methods

### Patients

A total of 65 patients (51W/14 M) with a definite diagnosis of Hashimoto’s thyroiditis (HT) were enrolled in the study as previously described.^21^ Twenty-three of them (16 W/7 M) showed the presence of an isolated thyroiditis (IT). Fifteen patients (11W/4 M) had HT and non-segmental *vitiligo* (VIT), 16 patients (15 W/1 M) had HT and chronic atrophic gastritis (CAG), and 11 patients (9 W/2 M) had HT and celiac disease (CD). All patients were spontaneously or pharmacologically euthyroid, but in the IT group, patients were further divided into euthyroid (14 patients, 12 W/2 M) and hypothyroid (9 patients, 4 W/5 M). Twenty healthy donors (HD) (16W/4 M) were also enrolled and served as an internal control. The anthropometric and functional characteristics are reported in Table 1.

**Table 1:** Healthy donors and patients’ characteristics

	*n*	W/M	Age	BMI	TSH mUI/L	FT4 ng/dL
HD	20	14/6	40 (30–53)	23,7 (23,3–24,7)	1,9 (1,4–2,5)	/
HT	65	51/14	50 (44–60)	24,9 (21,6–27,2)	2,4 (1,3–3,1)	1,1 (0,9–1,4)
VIT	15	11/4	55 (48–60)	23,8 (22,3–25,3)	1,5 (1–3,1)	1 (0,9–1,3)
CAG	16	15/1	58 (51–60)	26,6 (22,4–29,3)	2,1 (1–2,9)	1,3 (1–1,4)
CD	11	9/2	49 (33–60)	25,1 (21,4–25,4)	2 (1,2–2,7)	1,1 (0,9–1,3)
IT	23	16/7	45 (35–50)	23,3 (21,6–28,1)	2,9 (1,6–18,4)	0,9 (0,8–1,2)
EU	14	12/2	47 (44–54)	22,8 (21,6–28,4)	2,1 (1,4–2,8)	1,3 (1,1–1,4)
HYPO	9	4/5	36 (32–50)	24 (21,6–26,4)	51,3 (11,8–107,5)	0,5 (0,4–0,7)

The diagnosis of Hashimoto’s thyroiditis has been made based on the presence at least of two out of the following three criteria: 1) characteristic US features (hypoechogenity, dysomogeneity, pseudonodules, echogenic septae, etc.); 2) the presence of high anti-thyroperoxidase auto-antibodies (anti-TPOAb) titers; and/or 3) hypothyroidism.^22^ The diagnosis of euthyroid Hashimoto thyroiditis has been based on the presence of both the remaining criteria (i.e., high anti-TPOAb titers and typical US pattern).

The diagnosis of each associated disease was defined based on diagnostic criteria approved by the specific Consensus Conference and confirmed by serologic and histological tests, where required.^23–26^

All patients and healthy donors enrolled were negative for infection and/or inflammatory disorders in the last 6 months; patients with different but relevant chronic disorders (cancer, diabetes, COPB, obesity, and renal failure) and those who were pregnant and nursing and/or treated with drugs interfering with immune response (NSAIDs, steroids, immunosuppressant drugs, and immunomodulatory drugs) were positively excluded. All CD patients were in gluten-free diet for almost 6 months. All patients were informed about the purpose of the study and gave a written informed consent. The study has been approved by the local ethical committee (ASL C, Roma-Latina, Italy) according to the local ethical rules and to the guidelines of the Declaration of Helsinki.

### Methods

All patients gave fasting morning blood samples, collected in EDTA. CD20^+^ T lymphocytes were analyzed by flow cytometry with a staining no wash protocol. A volume of 100 μL of whole blood was incubated for 25 min on ice in the dark with the following directly conjugated antibodies: anti-CD4-APC-H7 (clone RPA-T4, 5 μL); anti-CD8-FITC (clone RPA-T8, 5 μL); anti-CD16-PE (clone 3G8, 2 μL); anti-CD19-PE (clone HIB19, 10 μL); anti-CD20-APC (clone 2H7, 10 μL); anti-CD45-PerCP (clone HI30, 5 μL); and anti-CD56-PE (clone B159, 10 μL). For the measurement of intrinsic cellular fluorescence (i.e., autofluorescence), 100 μL of whole blood was incubated in the same way without adding antibodies. At the end of the incubation, 1 mL of lysis buffer (BD Pharm Lyse, Becton Dickinson) was added to each sample and the samples were incubated for 15 min at room temperature, in the dark to allow the lysis of red blood cells which would interfere with the cytometric acquisition. At the end of this second incubation, the samples were directly acquired using a FACs ARIA II cell sorter (Becton Dickinson) equipped with a 488-nm solid-state laser and a 633-nm HeNe laser.

#### Gating strategy

Total T lymphocytes were identified as CD45- and CD3-positive cells. CD16 and CD19 were used to exclude B lymphocytes, while CD56 was used to exclude natural killer cells. Total T lymphocytes were further characterized as CD4 positive, CD8 positive, and double negative cells (CD4^−^/CD8^−^). Finally, for each of these subsets, the percentage of CD20-positive cells was analyzed.

#### Statistical analysis

Results were presented as median values. The difference between two groups was calculated using the non-parametric Mann–Whitney U test. When comparing more than two groups, the non-parametric Kruskal–Wallis test followed by the Dunn post-test was used to compare all pairs of data. INSTAT GraphPad Prism 9.0 software for Windows was used for the statistical analysis.

## Results

**Fig. 1:**
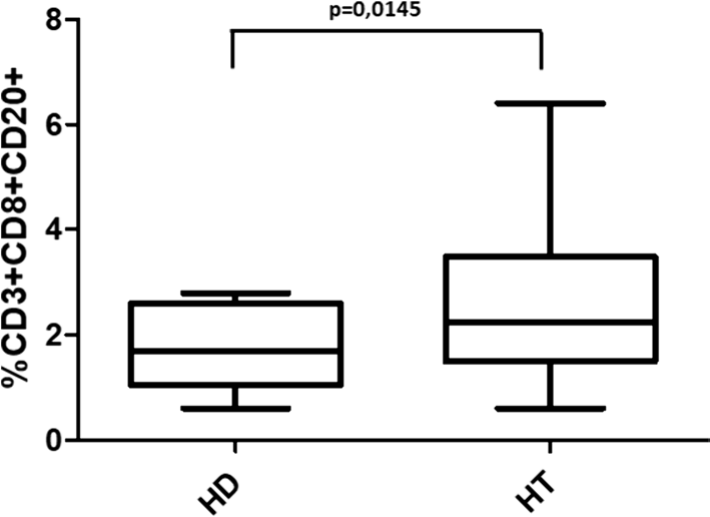
Percentages of CD3^+^CD8^+^CD20^+^ T cells in healthy donors (HD) and Hashimoto’s thyroiditis patients (HT)

The only difference in patients’ characteristics to be mentioned stems from age that was higher in patients with CAG than that in IT and in HD patients (*p* = 0.0091). This potential bias has been proven irrelevant (see below). Among IT patients, the age difference between euthyroid and hypothyroid groups was not statistically significant (*p* = 0.3486). Table 2 shows the median percentage of the lymphocytic subsets in HD patients and in patients with Hashimoto thyroiditis (HT). No difference emerged from the analysis of CD20^−^ T lymphocytes. However, among CD20^+^ T cell subsets, CD3^+^CD8^+^CD20^+^ cells were significantly higher in HT than those in HD group (*p* = 0.0145) (Fig. 1).

**Table 2:** Median percentage values (and relative IQR) of the lymphocytic subsets in healthy donors (HD) and Hashimoto’s thyroiditis patients (HT)

	% of CD3^+^ T lymphocytes		
	CD4^−^CD8^−^	CD4^+^	CD8^+^
HD	7.8 (6.8–13.8)	61 (58.3–68.9)	28.2 (22.5–33.6)
HT	8.2 (6.3–9.8)	63.1 (57.2–69.7)	27.4 (22.6–31.7)
*p *value	0.7487	0.4676	0.5443

	% of respective subset			
	CD20^+^	CD4^−^CD8^−^CD20^+^	CD4^+^CD20^+^	CD8^+^CD20^+^
HD	0.9 (0.7–1.4)	1.6 (1.2–2.2)	0.5 (0.4–0.6)	1.7 (1.0–2.6)
HT	1.1 (0.8–1.7)	1.4 (1.0–2.6)	0.5 (0.3–0.8)	2.2 (1.5–3.5)
*p *value	0.2628	0.9234	0.4700	**0.0145**

Bold indicates the *p* values reaching statistical significance (*p* < 0.05)

p value has been calculated using Mann–Whitney test

**Fig. 2:**
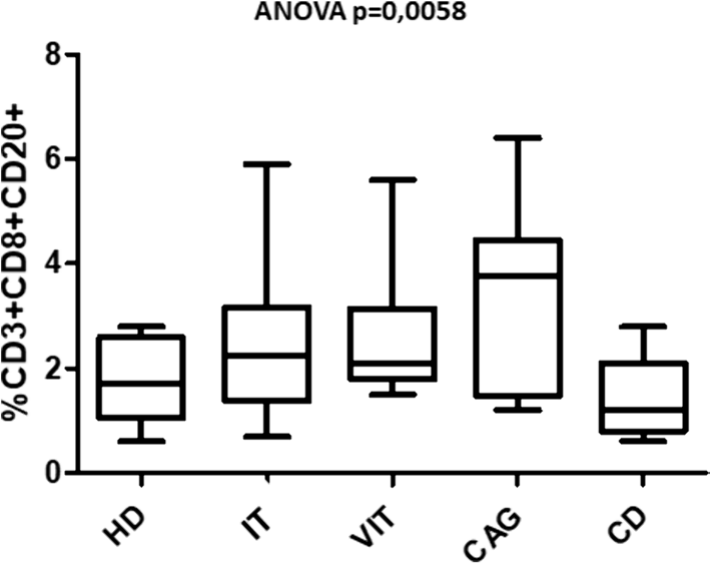
Percentages of CD3^+^CD8^+^CD20^+^ T cells in healthy donors (HD) and patients affected by Hashimoto’s thyroiditis presenting isolated (IT) or associated with vitiligo (VIT), chronic atrophic gastritis (CAG), celiac disease (CD)

The median percentages of the lymphocytic subsets in HD and in the HT patients when subdivided in the described four patients’ groups are shown in Table 3. When analyzing the canonical T lymphocytic subsets, it appears that CD3^+^CD8^+^ cells were significantly lower in CAG group than those in IT (*p* = 0.037). Since this specific subgroup had a significant older age than the one in IT group, a correlation was searched. The Spearman’s coefficient did not show any correlation between values of these cells and the age of patients (*p* = ns), dispelling the doubt of an age-related bias. Among CD20^+^ T lymphocyte subsets, CD3^+^CD8^+^CD20^+^ cells were also significantly higher in CAG than those in HD and CD groups (*p* = 0.0058) (Fig. 2). Again, Spearman’s coefficient did not show correlation between values of these cells and the age of patients (*p* = ns).

**Table 3:** Median percentage values (and relative IQR) of the lymphocytic subsets in healthy donors (HD) and patients affected by Hashimoto’s thyroiditis presenting isolated (IT) or associated with vitiligo (VIT), chronic atrophic gastritis (CAG), celiac disease (CD)

	% of CD3^+^ T lymphocytes		
	CD4^−^CD8^−^	CD4^+^	CD8^+^
HD	7.8 (6.8–13.8)	61.0 (58.3–68.9)	28.2 (22.5–33.6)
IT	8.4 (6.3–10.8)	62.5 (56.9–67.2)	28.1 (25.8–32.4)
VIT	7.2 (6–9.2)	61.9 (53.1–70.6)	30.5 (20.3–41.1)
CAG	9.0 (6.3–9.6)	67.2 (61.4–74.3)	23.6† (15.6–27)
CD	8.2 (6.9–15.1)	66.2 (54.5–67.5)	27.4 (25.6–29.5)
*p* value	0.7761	0.2170	0.037^**§**^

	% of respective subset			
	CD20^+^	CD4^−^CD8^−^CD20^+^	CD4^+^CD20^+^	CD8^+^CD20^+^
HD	0.9 (0.7–1.4)	1.6 (1.2–2.2)	0.5 (0.4–0.6)	1.7* (1–2.6)
IT	1.1 (0.7–1.5)	1.4 (1–2)	0.4 (0.3–0.7)	2.2 (1.4–3.2)
VIT	1.3 (1–1.7)	1.6 (1–2.3)	0.6 (0.4–0.8)	2.1 (1.8–3.1)
CAG	1.1 (0.9–1.9)	1.8 (1.1–3.2)	0.5 (0.3–0.9)	3.7* (1.5–4.4)
CD	1.1 (0.5–2.1)	1.2 (0.9–2.4)	0.4 (0.3–0.7)	1.2* (0.8–2.1)
p value	0.4383	0.9023	0.6168	0.0058^**§**^

^§^Kruskal–Wallis test

^§^Kruskal–Wallis test

^*^Dunn’s post test < 0.05 (CAG vs HD, CAG vs CD)

^†^Dunn’s post test < 0.05 (CAG vs IT)

**Fig. 3:**
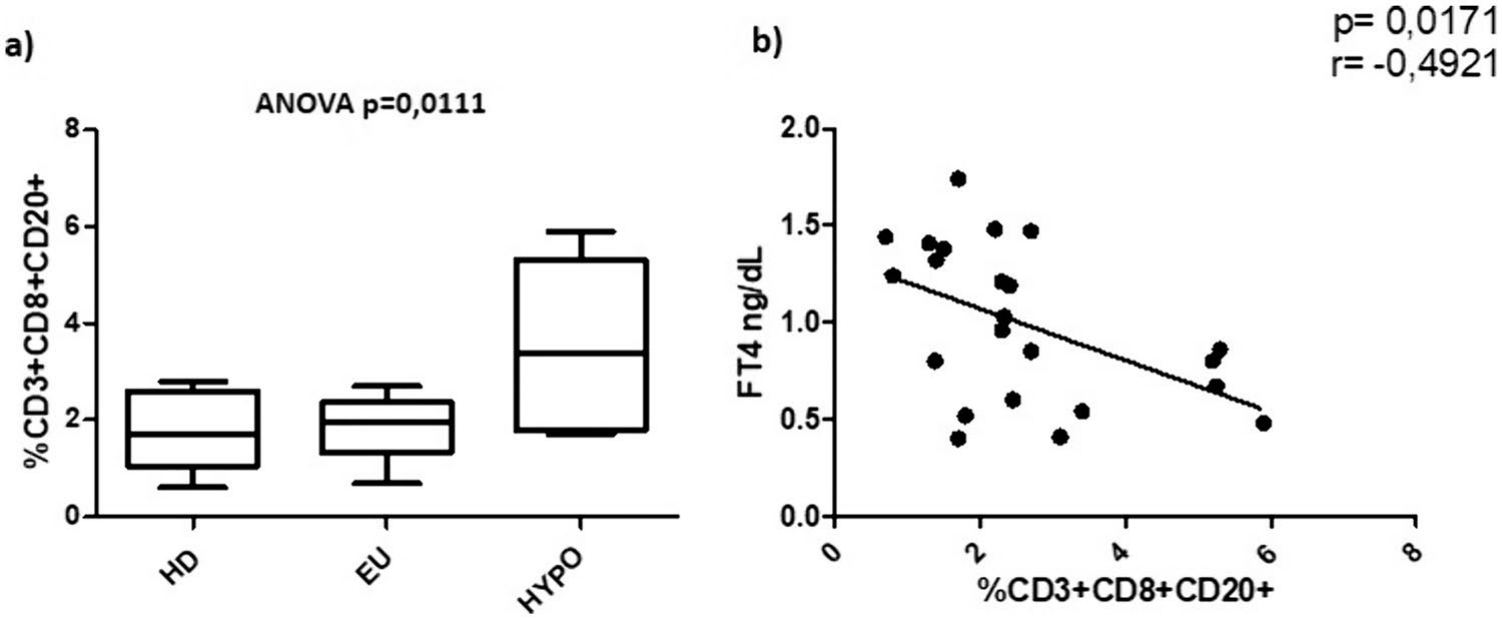
**a** Percentages of CD3^+^CD8^+^CD20^+^ T cells in in healthy donors (HD) and patients affected by isolated Hashimoto’s thyroiditis divided into euthyroid (EU) and hypothyroid one (HYPO). **b** correlation between CD3^+^CD8^+^CD20^+^ T cells and FT4 values in patients with isolated Hashimoto’s thyroiditis

Table 4 shows the median percentages of the lymphocytic subsets in HD and IT patients divided on the basis of thyroid function. Again, CD3^+^CD8^+^CD20^+^ population provides the most interesting results being significantly higher in hypothyroid group as compared with healthy donors and almost doubled as compared with euthyroid HT patients (*p* = 0.0111) (Fig. 3a). Spearman’s coefficient did not show correlation between values of these cells and TSH levels (*p* = ns) but showed a negative correlation between the percentages of these cells and the levels of FT4 (*p* = 0.0171; *r* = -0.4921) (Fig. 3b).

**Table 4:** Median percentage values (and relative IQR) of the lymphocytic subsets in healthy donors (HD) and patients affected by isolated Hashimoto’s thyroiditis divided into euthyroid (EU) and hypothyroid one (HYPO)

	% of CD3^+^ T lymphocytes		
	CD4^−^CD8^−^	CD4^+^	CD8^+^
HD	7.8 (6.8–13.8)	61 (58.3–68.9)	28.2 (22.5–33.6)
EU	8.6 (6.2–10.6)	62.5 (59.2–64.2)	28.6 (25.8–31.3)
HYPO	7.7 (7.3–12.7)	60.7 (51.7–69.7)	28.1 (26.1–35.3)
*p* value	0.7787	0.9734	0.7987

	% of respective subset			
	CD20^+^	CD4^−^CD8^−^CD20^+^	CD4^+^CD20^+^	CD8^+^CD20^+^
HD	0.9 (0.7–1.4)	1.6 (1.2–2.2)	0.5 (0.4–0.6)	1.7 ** (1.0–2.6)
EU	10 (0.8–1.1)	1.3 (1.0–1.6)	0.3 (0.3–0.6)	1.9 * (1.3–2.4)
HYPO	1.5 (0.7–2.1)	2.1 (0.9–3.6)	0.7 (0.3–0.8)	3.4 (1.8–5.3)
*p* value	0.2856	0.3991	0.8629	**0.0111**^**§**^

Bold indicates the *p* values reaching statistical significance (*p* < 0.05)

^§^Kruskal–Wallis test *Dunn’s post test < 0.05 EU vs HYPO *-

^**^Dunn’s post test < 0.01 HD vs HYPO**

## Discussion

The main findings of this pilot study stem from the difference observed between patients with HT and healthy donors as well as those recorded in the subgroups with poly-autoimmunity. Indeed, HT group showed a significant increase in the CD8^+^CD20^+^ T cell population as compared to healthy donors. These data are in keeping with the quantitative alterations that have already been described for other autoimmune diseases such as multiple sclerosis.^5^ CD20^+^ T cells produce large amounts of proinflammatory cytokines.^5^ It is not therefore surprising that they are increased in the group of patients suffering from autoimmune thyroiditis in which T-helper 17 polarization in the early phase and T-helper 1 polarization later on, and their related proinflammatory cytokines, play a prominent pathogenic role.^27–28^ Despite the reduction of CD3^+^CD8^+^ T cells in patients with CAG even in these patients, a significant increase of CD3^+^CD8^+^ CD20^+^ T cells has been observed. This result may be in keeping with the pathogenesis of atrophic gastritis in which the cellular damage is mainly a CD4^+^- rather than a CD8^+^-driven process.^29^ However, it is intriguing to note this clusterization of CD20^+^ T cells, possessing a high inflammatory profile.

The influence of autoimmune disorders associated with HT has been studied in different settings.^30–32^ In the present study, upon subdivision of HT patients in four groups, CD8^+^CD20^+^ T cells resulted significantly higher in patients with HT + CAG than those in HD and HT + CD groups. As patients in HT + CAG group showed older age and CD20^+^ cells’ percentage increase with age^33^, according to their memory phenotype, age itself may have represented a confounding factor. The doubt was dispelled since no correlation emerged between patient age and the percentage of these cells. Thus, this result appears to be specific of the association of thyroiditis and gastritis, and this hypothesis is strengthened by the fact that patients with HT + CD showed the lowest values of these cells, even compared to HD. Indeed, being all CD patients on gluten-free diet, their inflammatory status was typical of a quiescent state of the disease. This finding, being the exact role of these cells poorly characterized, suggests the anti-inflammatory role of gluten-free diet, not only at the gut level but even at a systemic one, probably involving the modulation of the CD8^+^CD20^+^ T cell subset.^34–35^ The potentially more important finding emerged when patients with isolated thyroiditis were divided based on their thyroid function. Once again, CD8^+^CD20^+^ T cell subset was significantly higher in hypothyroid group than that in the euthyroid one and in HD. Of course, any interpretation of this finding may appear speculative. However, two main hypotheses may help in explaining these data; the first one is that these cells are active in an early and highly destructive phase of autoimmune process, as confirmed by the fact that all hypothyroid patients with Hashimoto’s thyroiditis were newly diagnosed. As an alternative hypothesis, one could conceive that thyroid function may quantitatively affect these cells; indeed, in patients with isolated thyroiditis, no correlation with serum TSH emerged, while a negative correlation between these cells and FT4 levels has been observed. This correlation was not confirmed when the whole group of HT patients was analyzed (data not shown) probably owed to the little variations of FT4 in euthyroid patients and due to the effect of different associated autoimmune diseases on CD20^+^ T lymphocytes.

## Conclusions

This study has some limitations: being a pilot study, it encompasses a small number of patients and it lacks a functional analysis of the lymphocytic populations. Furthermore, the analysis of T lymphocytes was carried out on peripheral circulating cells, but the peculiar characteristics of peripheral lymphocytic population were clearly demonstrated even in organ-specific autoimmune disorders. ^36,37,38^ However, this study has been the first to characterize CD20^+^ T cell subsets in autoimmune thyroiditis. Our findings seem to indicate that CD8^+^CD20^+^ T lymphocytes are activated in patients with autoimmune thyroiditis and differently modulated according to thyroid function, the phase of the autoimmune process, and the autoimmune disorders associated with thyroiditis. An interesting future perspective could be the characterization and functional evaluation of CD20^+^ T cells in the whole spectrum of autoimmune thyroid disease.

## Data Availability

Data are available upon request.
